# The Acrylamide (S)-2 As a Positive and Negative Modulator of Kv7 Channels Expressed in *Xenopus laevis* Oocytes

**DOI:** 10.1371/journal.pone.0008251

**Published:** 2009-12-11

**Authors:** Sigrid Marie Blom, Nicole Schmitt, Henrik Sindal Jensen

**Affiliations:** 1 Section of Early Target Pharmacology and Physiology, H. Lundbeck A/S, Copenhagen, Denmark; 2 Department of Biomedical Sciences and Danish National Research Foundation Centre for Cardiac Arrhythmia, University of Copenhagen, Copenhagen, Denmark; 3 Section of Early Target Pharmacology and Physiology, H. Lundbeck A/S, Copenhagen, Denmark; University of Cincinnati, United States of America

## Abstract

**Background:**

Activation of voltage-gated potassium channels of the Kv7 (KCNQ) family reduces cellular excitability. These channels are therefore attractive targets for treatment of diseases characterized by hyperexcitability, such as epilepsy, migraine and neuropathic pain. Retigabine, which opens Kv7.2-5, is now in clinical trial phase III for the treatment of partial onset seizures. One of the main obstacles in developing Kv7 channel active drugs has been to identify compounds that can discriminate between the neuronal subtypes, a feature that could help diminish side effects and increase the potential of drugs for particular indications.

**Methodology/Principal Findings:**

In the present study we have made a thorough investigation of the Bristol-Myers Squibb compound (S)-N-[1-(4-Cyclopropylmethyl-3,4-dihydro-2*H*-benzo[1], [4]oxazin-6-yl)-ethyl]-3-(2-fluoro-phenyl)-acrylamide [(S)-2] on human Kv7.1-5 channels expressed in *Xenopus laevis* oocytes. We found that the compound was a weak inhibitor of Kv7.1. In contrast, (S)-2 efficiently opened Kv7.2-5 by producing hyperpolarizing shifts in the voltage-dependence of activation and enhancing the maximal current amplitude. Further, it reduced inactivation, accelerated activation kinetics and slowed deactivation kinetics. The mechanisms of action varied between the subtypes. The enhancing effects of (S)-2 were critically dependent on a tryptophan residue in S5 also known to be crucial for the effects of retigabine, (S)-1 and BMS-204352. However, while (S)-2 did not at all affect a mutant Kv7.4 with a leucine in this position (Kv7.4-W242L), a Kv7.2 with the same mutation (Kv7.2-W236L) was inhibited by the compound, showing that (S)-2 displays a subtype-selective interaction with in the Kv7 family.

**Conclusions/Significance:**

These results offer further insight into pharmacological activation of Kv7 channels, add to the understanding of small molecule interactions with the channels and may contribute to the design of subtype selective modulators.

## Introduction

Voltage-gated potassium (Kv) channels of the Kv7 (KCNQ) family are important regulators of neuronal excitability. Five members, Kv7.1-5, have been reported so far. Kv7.1 is best known for its expression in cardiac tissue where it assembles with KCNE1 to conduct the I_Ks_ current [1], [2]. The other four proteins are reported as the neuronal Kv7 channels (although they are also found in other tissues [3], [4]) and constitute the molecular correlates of the M-current [5], [6]. This current is primarily due to heteromeric assemblies of Kv7.2 and Kv7.3 [7], but also Kv7.4 and Kv7.5 show M-current characteristics [8]–[11]. All subtypes share the general structure of Kv channels with four subunits assembling into functional tetramers, either homo- or heteromeric. Each subunit comprises six transmembrane helices, S1-S6, and has a pore forming domain, which is formed by a P-loop between the fifth and sixth helix. The fourth helix forms the voltage sensor that contains several arginine residues and is therefore strongly positively charged [6].

Neuronal Kv7 channels activate at membrane potentials that are below the threshold required for generation of an action potential. They therefore allow for potassium flux that opposes depolarization, hence their activation will make the neuron less excitable. This makes them particularly interesting as targets for treatment of CNS diseases linked to hyperexcitability, including epilepsy, neuropathic pain and migraine. In addition, Kv7.2 and Kv7.4 are expressed in dopaminergic neurons of the midbrain, where they modulate the release of dopamine [12]–[15]. This has opened the possibility of Kv7 channels as a target for treatment of diseases characterized by dysfunction in the dopaminergic system, such as ADHD, drug abuse, and schizophrenia [15].

Negative and positive modulators of the M-channels have existed even before the molecular correlates were identified and cloned. Retigabine, the best described of these, is now in clinical trials phase III for the treatment of partial-onset seizures. Retigabine enhances the current through all neuronal homo- and heteromeric Kv7 channels, but not the cardiac Kv7.1. It induces a large hyperpolarizing shift in the voltage-dependence of activation of the channels, accelerates the activation and slows the deactivation kinetics [16]–[18]. Though several new classes of modulators have emerged since the cloning of Kv7 channels, there is still a lack of truly subtype selective compounds that can discriminate between the neuronal Kv7 channels. It is therefore still impossible to study the contribution of each individual subtype to the *in vivo* effect of e.g. retigabine. This knowledge would be valuable for identification of the ideal subtype to target for each indication.

**Figure 1:**
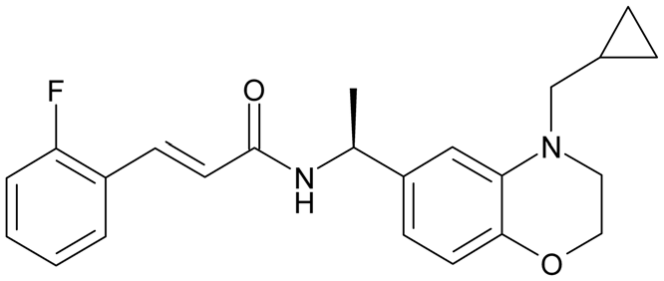
Chemical structure of (S)-2.

In the present study we thoroughly investigated the effects of the Bristol-Myers Squibb compound (S)-N-[1-(4-Cyclopropylmethyl-3,4-dihydro-2*H*-benzo[1], [4]oxazin-6-yl)-ethyl]-3-(2-fluoro-phenyl)-acrylamide [(S)-2] (fig. 1) on human Kv7.1-5 channels. The compound has previously been reported as a potent and efficacious Kv7.2 opener with significant activity in reducing hyperexcitability in rat hippocampal slices [19]. We show that (S)-2 is an activator of all neuronal Kv7 channels, but an inhibitor of Kv7.1. Furthermore, (S)-2 binds to the “retigabine-binding site”, however, while having no effect on a Kv7.4 channel with the critical mutation that renders the channel retigabine insensitive, (S)-2 inhibits a Kv7.2 channel with the same mutation, thereby revealing a subtype selectivity of modulation and interaction. In addition, our work indicates that in contrast to retigabine, (S)-2 is not dependent on being bound to all four subunits simultaneously for its enhancing effect, further discriminating (S)-2 from the pan-reactive action of retigabine.

## Materials and Methods

### Ethics Statement

The care of *Xenopus laevis* and the oocyte extraction procedure were performed according to national guidelines and approved by the Danish Animal Experiments Inspectorate.

### Molecular Biology

The point mutations W236L in Kv7.2 and G285S in Kv7.4 were introduced using mutated oligonucleotide extension (PfuTurbo Polymerase, Stratagene, La Jolla, CA, USA) from a plasmid template harboring the cDNA of interest, digested with DpnI (Fermentas, St. Leon, Germany) and transformed into *E.coli* XL1 Blue cells. The construct was verified by complete DNA sequencing of the cDNA insert. The Kv7.4-W242L mutant was a kind gift from Bo Hjorth Bentzen [20]. cRNA for injection was prepared from linearized human wild-type (WT) Kv7.1-5 and mutant Kv7.2 and Kv7.4 in pGEM-HE vectors using the T7 m-Message Machine kit (Ambion, Austin, TX, USA) according to the manufacturer's instructions. RNA concentrations were quantified using UV spectroscopy and RNA quality was checked by gel electrophoresis.

### Expression in *Xenopus laevis* Oocytes

Female *Xenopus laevis* were anaesthetized by immersion in a 0.4% (w/v) solution of 3-aminobenzoic acid ethyl ester (Sigma, St. Louis, Missouri, USA) for 15–20 min. Ovarian lobes were cut off through a small abdominal incision and subsequently defolliculated by enzymatic treatment with 0.5 mg/mL collagenase type IA (Sigma, St. Louis, MO, USA) in OR2 solution (in mM: 82.5 NaCl, 2 KCl, 1 MgCl_2_, 5 HEPES, pH 7.4) for 3 hours. Oocytes were then kept in Modified Barth's Saline (in mM: 88 NaCl, 1 KCl, 2.4 NaHCO_3_, 0.41 CaCl_2_, 0.82 MgSO_4_, 0.3 Ca(NO_3_)_2_, 15 HEPES, pH 7.4 suppl. with 100 U/mL penicillin and 100 µg/mL streptomycin) at 18°C until injection. cRNA was injected using a Nanoliter Injector (World Precision Instruments, Sarasota, Florida, USA). For Kv7.1 between 2 and 10 ng of cRNA was injected, for Kv7.2, Kv7.2-W236L and Kv7.5 10–25 ng was injected and for Kv7.4 and Kv7.4-W242L 3–6 ng was injected. For co-expression of Kv7.2 and Kv7.3 2 ng of each was injected. The oocytes were kept in Modified Barth's Saline at 18°C and currents were recorded after 2–7 days.

#### Electrophysiology

Kv7 currents in *Xenopus laevis* oocytes were recorded using two-electrode voltage-clamp. The recordings were performed at room temperature in Ringer buffer (in mM: 115 NaCl, 2.5 KCl, 1.8 CaCl_2_, 0.1 MgCl_2_, 10 HEPES, pH 7.4) using an Axon GeneClamp 500B two-electrode voltage-clamp amplifier (Axon Instruments Inc., Union City, CA, USA) and a Digidata 1440A digitizer (Axon Instruments). The oocytes were placed in homemade perfusion chambers connected to a continuous flow system. Recording electrodes were pulled from filamented borosilicate glass capillaries (type GC150TF-10, Harvard Apparatus, Kent, U.K.) on a horizontal Flaming/Brown Model P-97 micropipette puller (Sutter Instrument Company, Novato, California, USA) and filled with 1 M KCl. The electrodes had a resistance of 0.5–2.5 MΩ. All experiments were performed on minimum two batches of oocytes.

#### Rubidium Flux Assay

Chinese hamster ovary (CHO) cells were transiently transfected with human Kv7.2, Kv7.2-W236L or Kv7.4 cDNA inserted into pcDNA3 vectors using Lipofectamine2000 and FuGENE 6, plated in 96 wells plates and incubated with 1 µCi/mL ^86^Rb overnight. The following day the cells were washed twice with assay buffer (HBSS with 20 mM HEPES, pH 7.4) and stimulated with (S)-2 diluted in stimulation buffer (assay buffer with 15 or 30 mM KCl for assessing activation or inhibition, respectively) for 30 min at 37°C. The supernatant was transferred to a new 96 well plate and added 100 µL scintillation fluid (Optiphase “Supermix”, PerkinElmer). The cells were washed, added 50 µL of assay buffer and scintillation fluid and incubated at room temperature at a shaker for 15 min to lyse the cells. The plates were finally counted on a liquid scintillation counter (Wallac MicroBeta® TriLux, PerkinElmer). Raw data was obtained as counts per minute (cpm) and are presented as cpm in the supernatant divided with total counts (supernatant+cell pellet) normalized using the equation (X-min)/(max-min)*100 where min and max are the cpm_sup_/cpm_total_ ratios obtained when stimulating with 15 and 100 mM KCl, respectively (for experiments with Kv7.2 and Kv7.4). Data from experiments with Kv7.2-W236L are normalized by dividing with the cpm_sup_/cpm_total_ ratio obtained when stimulating with 30 mM KCl.

### Drugs

(S)-2 was dissolved in dimethyl sulfoxide to obtain a concentrated stock solution. On the day of experiments the stock solution was thawed and diluted in Ringer buffer to the final concentrations. A concentration of 10 µM (S)-2 was used unless indicated otherwise. The final dimethyl sulfoxide concentration never exceeded 0.15% for experiments on oocytes and 1% for experiments on CHO cells. (S)-2 was synthesized in-house at H. Lundbeck A/S, Valby, Denmark, according to literature methods [19].

### Statistical Analysis and Curve Fitting

Data was acquired using pCLAMP 10.2 software (Molecular Devices, CA, USA) and analyzed using pCLAMP 10.2 and GraphPad Prism 4.0 (GraphPad Software Inc., CA, USA).

The voltage-dependence of activation was determined from tail current analysis using the current measured immediately after the transient capacitative current after stepping to −120 mV from potentials between −100 mV and +60 mV. Data was normalized to extend from 0–1 and the tail current-voltage relationship was fitted to the Boltzmann equation:
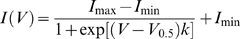
(Equation 1)where *I*_max_ is the maximum tail current, *I*_min_ is the minimum tail current, V_0.5_ is the potential for half maximal activation and *k* is the slope factor. A V_0.5_ was calculated for each individual experiment and statistical significance was estimated by two-tailed Student's *t*-test.

Activation kinetics was determined by eliciting currents from a holding potential of −80 mV to potentials between −100 mV and +60 mV of 5 s duration. An *F* test was used to assess whether single or double exponential functions gave the highest R^2^ value. For Kv7.2 and Kv7.2-W236L current traces were best fitted to a double exponential function:
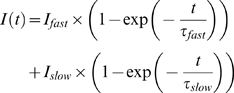
(Equation 2)where *I(t)* is the current at time *t*, *I_fast_* and *I_slow_* are the current amplitudes at infinite times, and *τ_fast_* and *τ_slow_* are the time constants of the fast and slow components, respectively.

The current traces for Kv7.4 were best fitted to a single exponential function:
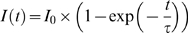
(Equation 3)where *I(t)* is the current at time *t*, *I*_0_ is the peak current and τ is the activation time constant.

For Kv7.2 and Kv7.2-W236L current traces between −40 mV and +40 mV were used fitting while for Kv7.4 current traces between −20 mV and +60 mV were used.

For investigation of deactivation kinetics oocytes were held for 5 s at +40 mV before stepping to potentials between −110 mV and −80 mV for 6 s. Tail current traces were best fitted to a double exponential function (Eq. 2). For data concerning activation and deactivation kinetics statistical significance was determined by two-way ANOVA followed by Bonferroni post-tests (if the overall P-value of the drug factor was less than 0.05). Before the statistical analyses of the fast component of the activation for Kv7.2 and Kv7.2-W236L the measured time constants were log-transformed to meet the assumption of normality. For traces fitted to double exponential functions only those where the sum of *I_fast_* and *I_slow_* equalled the peak current were included in the analysis.

Dose-response curves from electrophysiological experiments were made by plotting the increase in steady state current at 0 mV expressed in percentages as a function of drug concentration. The data were then analyzed by non-linear regression and fitted to the equation for sigmoidal dose-response with variable slope:
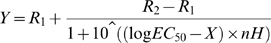
(Equation 4)where *R_1_* is the initial response, *R_2_* is the maximum response, *X* is the logarithm of the drug concentration and *nH* is the slope (Hill coefficient) of the curve.

To determine the EC_50_ or IC_50_ values from Rubidium flux experiments data from different test days were pooled, analyzed by non-linear regression and fitted to equation 4 (in the case of Kv7.2-W236L the parameter EC_50_ is replaced with IC_50_). pEC_50_ values and Hill slopes were calculated for each individual experiment and statistical significance was estimated by two-tailed Student's *t*-test.

For analysis of the remaining data statistical significance was determined by two-tailed Student's *t*-test if single comparisons were made and by two-way ANOVA followed by Bonferroni post-tests if multiple comparisons were made. Statistical analyses were carried out using GraphPad Prism 4.0. P<0.05 was accepted for statistical difference. All values are shown as mean±S.E.M.

## Results

### Effect of (S)-2 on Kv7.1-5 Current Amplitude

**Figure 2:**
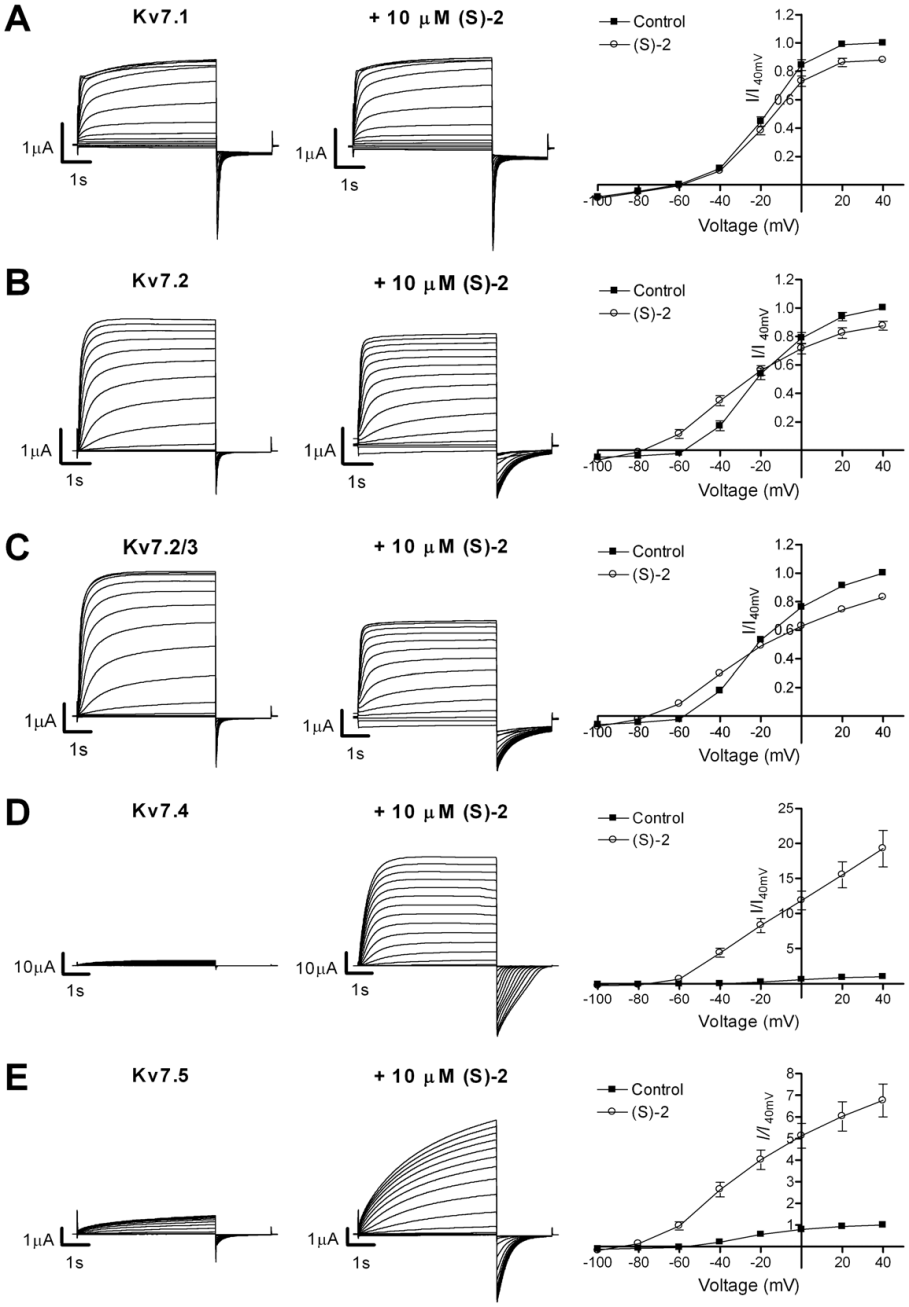
Activation of Kv7 channels by (S)-2. Representative two-electrode voltage-clamp current traces in the absence (left) and presence (middle) of 10 µM (S)-2 and effect of (S)-2 on current-voltage (I-V) relationship (right) of Kv7.1 (A), Kv7.2 (B), Kv7.2/Kv7.3 (C), Kv7.4 (D) and Kv7.5 (E) channels expressed in *Xenopus laevis* oocytes. The channels were activated by 5 s voltage steps from −80 mV to potentials ranging from –100 to +40 mV in 10 mV increments followed by a 2 S step to –120 mV. The steady state peak current amplitudes in the absence and presence of 10 µM (S)-2 were normalized against the current at +40 mV in control recordings and plotted against the test potential to obtain I-V curves (left). Bars represent S.E.M. and *n* = 6-17. Please note that in some instances the S.E.M. is so small that the error bars are not visible.

The effect of (S)-2 on Kv7 channels expressed in *Xenopus laevis* oocytes was studied using two-electrode voltage-clamp. The channels were activated by 5 s voltage steps from –80 mV to potentials ranging from –100 to +40 mV in 10 mV increments followed by a 2 s step to –120 mV in the absence (fig. 2, left panel) and presence (fig. 2, middle panel) of (S)-2. For current-voltage (I-V) curves, the steady state current was measured and plotted against the corresponding potential (fig. 2, right panel). On Kv7.1 application of (S)-2 led to a small but significant reduction in current at −20 mV and above (P<0.01, *n* = 10) (fig. 2A). The current amplitude at +40 mV in the presence of (S)-2 was 87.9±2.2% of control. On Kv7.2 channels (S)-2 exerted opposite effects on current amplitude at high and low voltages with the effect crossing over from positive to negative at –20 mV (fig. 2B). At –40 mV the current was 208.7±22.2% of control, while at +40 mV the current amplitude was 87.5±2.6% of control (*n* = 16). A similar effect was observed on heteromeric Kv7.2/3 channels (fig. 2C), where the current amplitude was 182.6±16.2% and 82.9±1.9% of control at –40 mV and +40 mV, respectively (*n* = 16). On Kv7.4 (fig. 2D) and Kv7.5 (fig. 2E) (S)-2 induced a profound increase in current amplitude at all potentials. The current amplitude at +40 mV was 1926.9±259.5% of control for Kv7.4 (*n* = 10) and 676.0±83.5% of control for Kv7.5 (*n* = 6).

### Effect of (S)-2 on Voltage-Dependence of Activation

**Figure 3:**
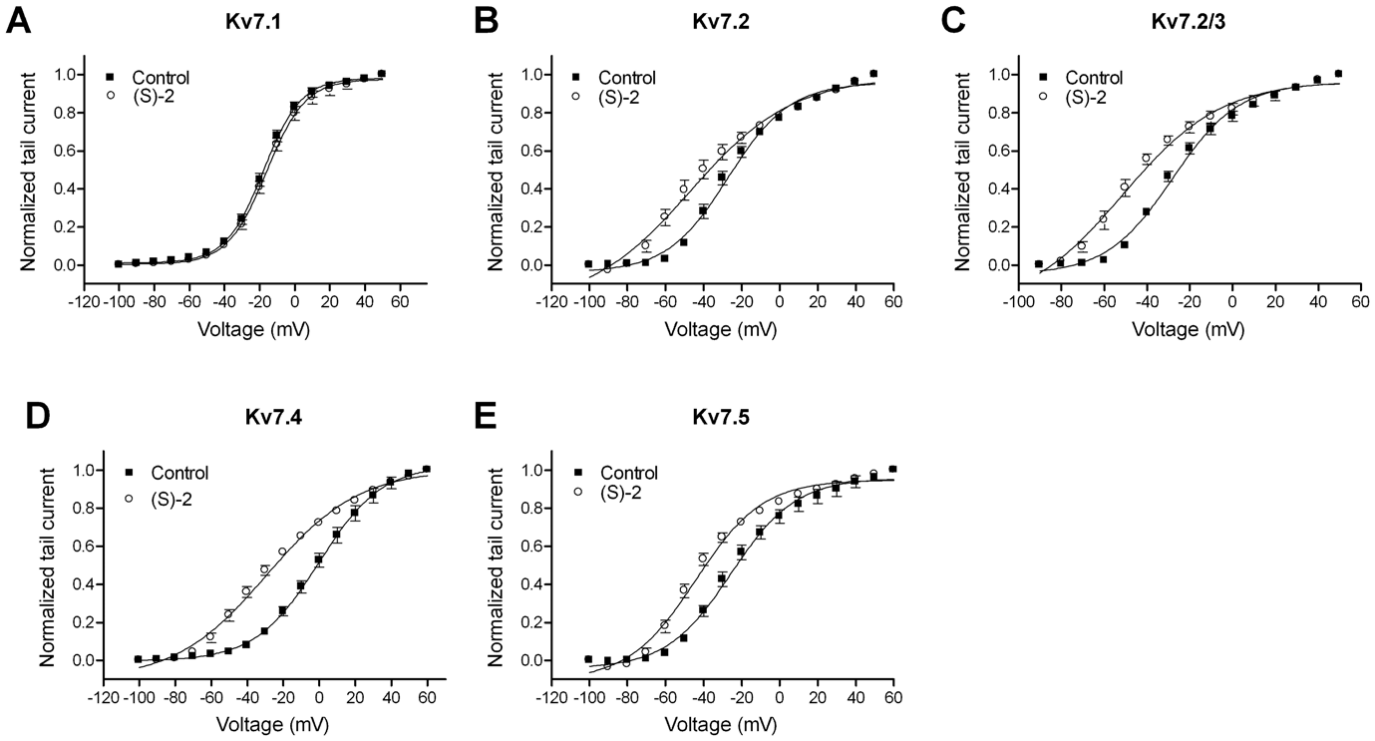
Effect of (S)-2 on the voltage-dependence of activation. Tail currents measured for Kv7.1 (A), Kv7.2 (B), Kv7.2/Kv7.3 (C), Kv7.4 (D) and Kv7.5 (E) after stepping back to −120 mV from 5 s long potentials between –100 and +60 mV were normalized and plotted against the preceding potential in the absence and presence of 10 µM (S)-2. The tail current-voltage relationship was then fitted to the Boltzmann equation (Eq. 1) to yield half-activation potentials (V_0.5_). Bars represent S.E.M. and *n* = 5–11.

The I-V curves showed a negative shift in the threshold for activation for the neuronal Kv7 channels upon (S)-2 application. To analyze the voltage-dependence of Kv7.1-5 activation, the tail-current measured after repolarization to –120 mV was normalized to extend from 0 to 1, plotted as a function of the preceding potential and fitted to a Boltzmann equation (Eq. 1, fig. 3). Under control conditions, Kv7.1 (fig. 3A) demonstrated a voltage-dependent activation with half-maximal activation (V_0.5_) of –18.0±1.6 mV. Application of (S)-2 did not result in a change of voltage-dependence (V_0.5_ with (S)-2: 16.0±1.4 mV, P = 0.4, *n* = 11). The voltage-dependence of activation of Kv7.2 (fig. 3B) was shifted significantly in the hyperpolarized direction by (S)-2 with a shift in V_0.5_ of 19.0 mV from –27.3±2.4 to –46.3±5.1 mV (P = 0.003, *n* = 10). Heteromeric Kv7.2/3 channels (fig. 3C) demonstrated a similar leftward shift in voltage-dependence after application of (S)-2 with a change in V_0.5_ of 18.0 mV from –26.4±2.3 mV to –44.4±5.6 mV (P<0.02, *n* = 5). The largest shift in V_0.5_ was observed for Kv7.4 (fig. 3D) where application of (S)-2 led to negative shift of 30.4 mV from –0.6±3.5 to –31.0±3.8 mV (P<0.0001, *n* = 11). For Kv7.5 (fig. 3E) there was a leftward shift in V_0.5_ of 18.1 mV from –25.1±2.6 mV to –43.2±2.4 mV (P = 0.0009, *n* = 5). Because of the great disparity between the effect of (S)-2 on the I-V relationship of Kv7.2 and Kv7.4, we continued with a more detailed examination of the effects on these two subtypes.

### Potency of (S)-2 on Kv7.2 and Kv7.4

**Figure 4:**
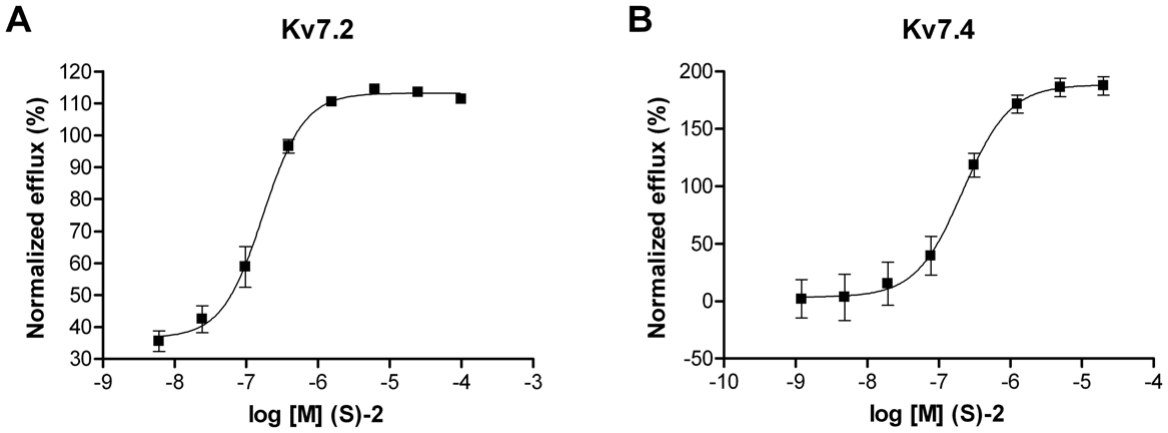
Potency of (S)-2 on Kv7.2 and Kv7.4. Dose-response relationship for the effect of (S)-2 on (A) Kv7.2 (*n* = 6) and (B) Kv7.4 (*n* = 8) measured using an ^86^Rb-flux assay.

To determine the potency of (S)-2 on Kv7.2 and Kv7.4 we performed a dose-response experiment using an ^86^Rb-flux assay. CHO cells expressing Kv7.2 or Kv7.4 were stimulated with increasing concentrations of (S)-2 and the normalized efflux of ^86^Rb was plotted against the corresponding drug concentration. Kv7.2 was activated by the compound with an EC_50_ of 0.17 µM (pEC_50_ = 6.77±0.05 M, *n* = 6, fig. 4A) corresponding well with the 0.06 µM reported previously [19]. Kv7.4 was activated by (S)-2 with an EC_50_ of 0.22 µM (pEC_50_ = 6.66±0.11 M, *n* = 8, fig. 4B).

### Effect of (S)-2 on Resting Membrane Potential

Expression of Kv7 channels in oocytes leads to a hyperpolarization of the oocytes to membrane potentials that lie close to the activation threshold of the channels. For example, oocytes that express Kv7.2/3 have a membrane potential of approximately −60 mV, which corresponds to the threshold for activation of Kv7.2/3. Application of retigabine leads to a further hyperpolarization of the membrane potential to a potential that corresponds to the activation threshold for the channel in the presence of retigabine [17]. We studied the effect of (S)-2 on the membrane potential of oocytes expressing either Kv7.2 or Kv7.4 using membrane potential recordings (data not shown). Oocytes expressing Kv7.2 had an average resting membrane potential of –54.3±1.2 mV. Application of 10 µM (S)-2 led to a rapid and significant hyperpolarization of the membrane potential to an average of –82.8±1.5 mV (P<0.001, *n* = 12). Similarly, application of (S)-2 to oocytes expressing Kv7.4 led to a ∼32 mV hyperpolarization of the membrane potential from –38.8±2.2 mV to –70.6±2.1 mV (P<0.001, *n* = 9).

### Effect of (S)-2 on Kv7 Channel Kinetics

**Figure 5:**
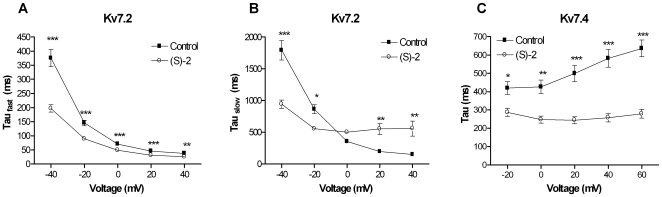
Effect of (S)-2 on the activation kinetics of Kv7.2 and Kv7.4. For determination of activation kinetics, current traces recorded during 5 s voltage steps from –80 mV to potentials between –40 and +40 mV for Kv7.2 in the absence and presence of (S)-2 were fitted to a double exponential function and the time constants τ_fast_ (A) and τ_slow_ (B) obtained were plotted against the step potential. For Kv7.4 current traces recorded during 5 s voltage steps to potentials between –20 and +60 mV were fitted to a single exponential function and τ is plotted against the step potential (C). Asterisks indicate statistical significant difference between absence and presence of (S)-2 determined by two-way ANOVA followed by Bonferroni post-test. * P<0.05, ** P<0.01 and *** P<0.001. Bars represent S.E.M. and *n* = 8-13.

From the current traces in figure 2 it is obvious that (S)-2 affected the kinetics of the neuronal Kv7 channels, most evidently the deactivation. To examine and quantify this further we calculated the time constants for activation and deactivation of Kv7.2 and Kv7.4 in the absence and presence of (S)-2. For Kv7.2 activation traces recorded after stepping from −80 mV to potentials between −40 mV and +40 mV were best fitted to a double exponential function (Eq. 2) that gives separate time constants for the slow and the fast component of the activation. Both the fast and the slow component of the activation kinetics for Kv7.2 showed a voltage-dependent relationship with very slow activation at −40 mV (τ_fast_ = 375.7±31.8 ms and τ_slow_ = 1792.5±162.0 ms, *n* = 13) and accelerated activation at more depolarized potentials (τ_fast_ = 37.6±5.7 ms and τ_slow_ = 149.1±11.8 ms at +40 mV, *n* = 13, fig. 5A and B). In the presence of (S)-2 the fast component of the activation was still voltage-dependent but the time course was significantly accelerated (τ_fast_ = 196.5±13.8 ms at −40 mV, P<0.001 and 25.5±1.8 ms at +40 mV, P<0.01, *n* = 10–13). Similar to how (S)-2 affected the current amplitude of Kv7.2, there was a cross-over in the effect on the slow component of the activation, here observed at around −10 mV. Below this voltage τ_slow_ was significantly decreased (τ_slow_ = 942.1±62.6 ms at −40 mV, *n* = 10–13, P<0.001) while it was significantly increased at potentials above 0 (τ_slow_ = 557.7±124.3 ms at +40 mV, P<0.01).

The activation traces recorded for Kv7.4 between −20 and +60 mV were best fitted to a single exponential function (Eq. 3). In the absence of drug the activation kinetics of Kv7.4 were slightly voltage-dependent with slower time course of activation at higher potentials ranging from 419.3±34.6 ms at –20 mV to 636.3±45.6 ms at +60 mV (*n* = 8, fig. 5C). In the presence of (S)-2 the activation kinetics became significantly accelerated and were largely voltage-independent with activation time constants of 286.3±22.5 ms at –20 mV (*n* = 8, P<0.05) and 278.9±24.4 ms at +60 mV (*n* = 8, P<0.001).

**Figure 6:**
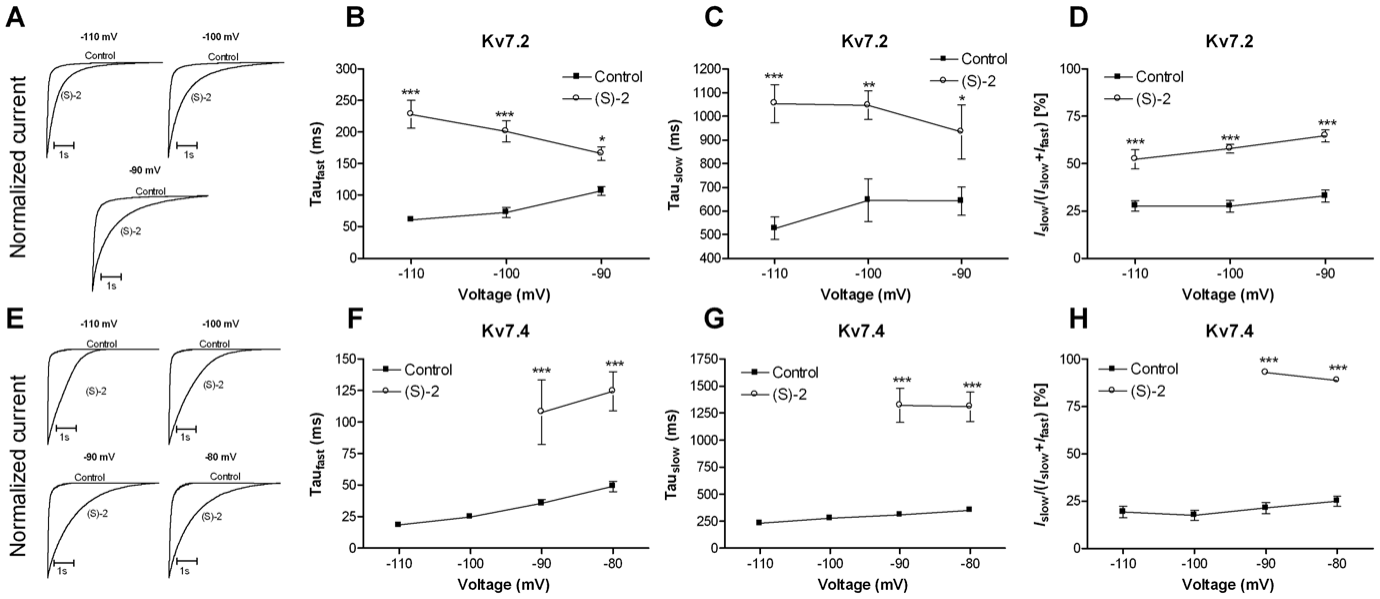
Effect of (S)-2 on the deactivation kinetics of Kv7.2 and Kv7.4. Normalized tail current traces for Kv7.2 (A) and Kv7.4 (E) obtained at the indicated potentials in the absence and presence of (S)-2 illustrating the pronounced effect of the compound on the deactivation kinetics. Deactivation kinetics for Kv7.2 were determined by fitting the tail currents measured at potentials between –110 mV and –90 mV after an activating step to +40 mV to a double exponential function. The time constants τ_fast_ (B) and τ_slow_ (C) were plotted against the potential. (D) Relative contribution of the slow component of the deactivation kinetics for Kv7.2. Tail currents measured between −110 and −80 mV for Kv7.4 were fitted to a double exponential function and τ_fast_ (F) and τ_slow_ (G) were plotted against the potential. (H) Relative contribution of the slow component of the deactivation kinetics for Kv7.4. Asterisks indicate statistical significant difference between absence and presence of (S)-2 determined by two-way ANOVA followed by Bonferroni post-test. * P<0.05, ** P<0.01 and *** P<0.001. Bars represent S.E.M. and *n* = 5–14.

Deactivation kinetics were studied using a voltage-clamp protocol that comprised a 5 s activation step to +40 mV followed by a 6 s test pulse to potentials between −110 mV and −80 mV. The deactivation traces were best fitted to a double exponential function (Eq. 2) for both Kv7.2 and Kv7.4. For Kv7.2 we did not observe a clear inward tail current after a step from +40 mV to −80 mV and this potential was therefore excluded. Normalized traces illustrating the effect of (S)-2 on the deactivation kinetics of Kv7.2 at −110, −100 and −90 mV is shown in fig. 6A. The time constants for the fast and the slow component of the deactivation for Kv7.2 in the absence and presence of (S)-2 are listed in table 1. (S)-2 significantly slowed both the fast and the slow component of the deactivation (fig. 6B and C). In addition, the compound significantly increased the relative contribution of the slow component at all potentials tested (fig. 6D).

**Table 1:** Deactivation kinetics.

	V[mV]	τ_slow_ [ms]	τ_slow_ [ms]	τ_fast_ [ms]	τ_fast_ [ms]	I_slow_/(I_slow_+I_fast_)	I_slow_/(I_slow_+I_fast_)
(S)-2		−	+	−	+	−	+
Kv7.2	−110	526.5±50.4 (*n* = 10)	1054.3±84.6 *** (*n* = 14)	61.2±4.8 (*n* = 10)	228.2±23.1 *** (*n* = 14)	27.6±2.8% (*n* = 10)	52.3±5.2% *** (*n* = 14)
	−100	646.0±93.4 (*n* = 14)	1047.3±62.5 * (*n* = 14)	72.8±8.3 (*n* = 14)	201.2±17.2 *** (*n* = 14)	27.5±3.1% (*n* = 14)	57.9±2.3% *** (*n* = 14)
	−90	642.3±62.2 (*n* = 12)	935.0±118.5 ** (*n* = 14)	106.7±7.1 (*n* = 12)	165.8±11.0 ** (*n* = 14)	33.0±3.4% (*n* = 12)	64.8±3.3% *** (*n* = 14)
Kv7.4	−110	231.4±14.4 (*n* = 9)	n.d., see text	18.3±1.0 (*n* = 9)	n.d., see text	19.2±3.0% (*n* = 9)	n.d., see text
	−100	276.6±22.3 (*n* = 10)	n.d., see text	24.9±1.7 (*n* = 10)	n.d., see text	17.5±2.8% (*n* = 10)	n.d., see text
	−90	308.5±23.6 (*n* = 10)	1320.6±176.4 *** (*n* = 5)	35.6±3.1 (*n* = 10)	107.8±28.5 *** (*n* = 5)	21.3±3.2% (*n* = 10)	93.0±0.7% *** (*n* = 5)
	−80	350.3±25.7 (*n* = 8)	1309.0±149.0 *** (*n* = 6)	49.0±4.2 (*n* = 8)	124.3±16.9 *** (*n* = 6)	25.0±2.9% (*n* = 8)	88.8±1.3% *** (*n* = 6)

Asterisks indicate statistical significant difference between absence and presence of (S)-2 determined by two-way ANOVA followed by Bonferroni post-test.

* P<0.05,

** P<0.01 and *** P<0.001.

Normalized traces illustrating the effect of (S)-2 on the deactivation kinetics of Kv7.4 is shown in fig. 6E. The time constants for the fast component of deactivation for Kv7.4 I the absence and presence of (S)-2 are listed in table 1. (S)-2 dramatically affected the deactivation kinetics of Kv7.4. At −110 mV and −100 mV the traces could no longer be fitted satisfactorily to an exponential function and instead showed an almost linear relationship. At −90 and −80 mV where satisfactory fits could be obtained both τ_fast_ and τ_slow_ were significantly increase (fig. 6F and G). This was accompanied by a dramatic increase in the relative contribution of the slow component of deactivation from ∼20% to ∼90% (fig. 6H).

### (S)-2 Affects the Inactivation of Kv7.4

**Figure 7:**
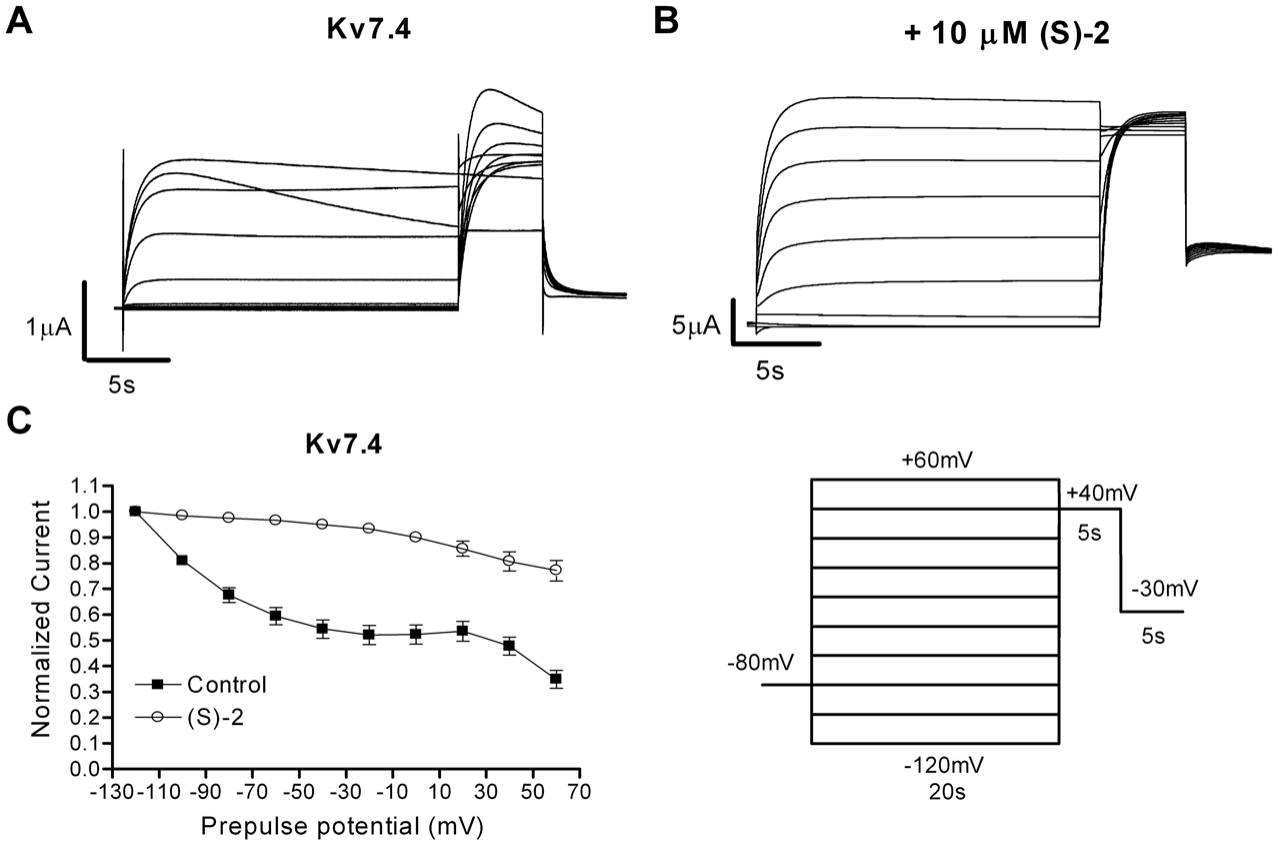
Effect of (S)-2 on the inactivation of Kv7.4. Representative two-electrode voltage-clamp recordings elicited by the voltage protocol shown in the inset in the absence (A) and presence (B) of 10 µM (S)-2. (C) The degree of inactivation in the absence and presence of (S)-2 is revealed by plotting the current amplitude measured at +40 mV normalized to the level measured after a prepulse potential of –120 mV as a function of the preceding prepulse potential. Bars represent S.E.M. and *n* = 11–14.

Although the M-current traditionally is described as non-inactivating, Kv7.4 and Kv7.5 have recently been found to inactivate [21], [22]. This inactivation can be modulated by chemical compounds like BMS-204352 [21]. To study the effect of (S)-2 on inactivation of Kv7.4 oocytes were clamped for 20 s at potentials between −120 mV and +60 mV in 20 mV increments. This step was followed by a 5 s activation step to +40mV and ended by a 5 s step to –30 mV. The peak current after the step to +40 mV was plotted against the preceding potential (V_pre_) and normalized to the peak current measured after a V_pre_ of –120 mV. This revealed a voltage-dependent inactivation for Kv7.4 with a maximal inactivation of 65.1±3.6% after a V_pre_ of +60 mV (*n* = 11, fig. 7A and C), which corresponds well with our previous results [21]. Application of (S)-2 resulted in a complete change in the inactivation properties of Kv7.4 with almost no inactivation at negative V_pre_ potentials and a maximal inactivation of 22.9±4.2% after a V_pre_ to +60 mV (*n* = 14, fig. 7B and C).

### (S)-2 Binds to the “Retigabine-Binding Site”

**Figure 8:**
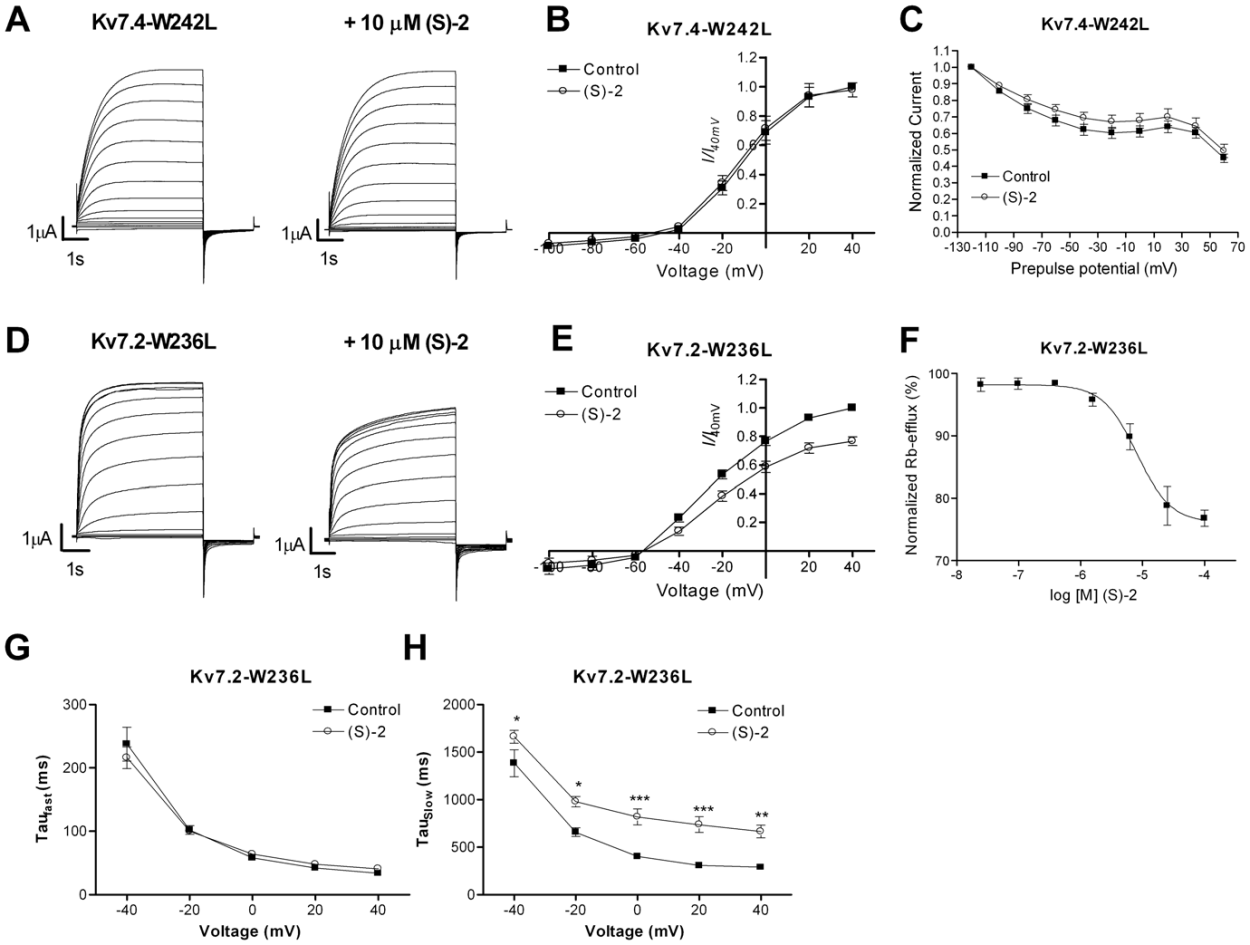
Dependency of (S)-2 on a tryptophan residue in S5. Representative two-electrode voltage-clamp current traces of Kv7.4-W242L (A) and Kv7.2-W236L (D) in the absence (left) and presence (right) of 10 µM (S)-2. Currents were elicited by 5 s depolarizing pulses between −100 and +40 mV in 10 mV increments from a holding potential of –80 mV followed by a 2 s step to –120 mV. The steady state peak current amplitudes of Kv7.4-W242L (B) and Kv7.2-W236L (E) in the absence and presence of 10 µM (S)-2 were normalized against the current at +40 mV in control recordings and plotted against the test potential to obtain current-voltage (I-V) curves (*n* = 10). (C) Cells expressing Kv7.4-W242L were investigated for inactivation by the same voltage protocol as in fig. 7. The plot shows the current amplitude measured at +40 mV normalized to the level measured after a prepulse potential of –120 mV as a function of the preceding prepulse potential, *n* = 11. (F) Dose-response relationship of the effect of (S)-2 on Kv7.2-W236L measured using a Rubidium-flux assay (*n* = 4). Effect of (S)-2 on the fast (G) and slow (H) components of the activation kinetics of Kv7.2-W236L. Current traces recorded during voltage steps from –80 mV to potentials between –40 and +40 mV in the absence and presence of (S)-2 were fitted to a double exponential function and the time constants τ_fast_ and τ_slow_ obtained were plotted against the step potential (*n* = 7). * P<0.05, ** P<0.01 *** P<0.001 determined by two-way ANOVA followed by Bonferroni post-test. Bars represent S.E.M.

Activation of Kv7.2-5 by retigabine is critically dependent on a conserved tryptophan residue in S5 of the channels [23], [24]. This residue has also been found to be part of the binding sites for BMS-204352 and (S)-1 [20] and has been suggested to be a major structural element of a more common Kv7 channel activator site. We investigated if the effect of (S)-2 also is dependent on this residue by substituting the tryptophan residue in Kv7.2 and Kv7.4 with a leucine. The resulting mutant channels, Kv7.2-W236L and Kv7.4-W242L, were then expressed in oocytes and exposed to (S)-2. For Kv7.4 the mutation resulted in a complete loss of sensitivity to (S)-2 (fig. 8A–C). Surprisingly, Kv7.2-W236L remained sensitive to (S)-2, but instead of activating the channel the compound induced a significant reduction in current amplitude at potentials from –20 mV and above (P<0.01, *n* = 10, fig. 8D and E). The current amplitude at +40 mV in the presence of (S)-2 was 76.6±3.2% of control. (S)-2 had no effect on the voltage-dependence of activation for either mutant (data not shown).

The inhibitory action of (S)-2 on Kv7.2-W236L was further analyzed by performing a dose-response experiment using an ^86^Rb-flux assay (fig. 8F). CHO cells expressing Kv7.2-W236L were stimulated with buffer containing 30 mM K^+^ (to activate the channels) in the presence of concentrations of (S)-2 between 0.024 µM and 100 µM. The normalized efflux of ^86^Rb was plotted against (S)-2 concentration and fitted to the Hill equation (Eq. 4). (S)-2 dose-dependently inhibited the efflux through Kv7.2-W236L with an IC_50_ of 8.1 µM (pIC_50_: 5.1±0.1 M, *n* = 4).

As seen in fig. 8A (S)-2 affected the activation kinetics of the mutated Kv7.2, hence we calculated activation time constants for the current traces at potentials between −40 mV and +40 mV (fig. 8G and H). Like for the WT Kv7.2 the activation traces of Kv7.2-W236L were best fitted to a double exponential function (Eq. 2). (S)-2 did not affect the fast component of the activation (τ_fast_ = 237.8±26.7 ms at –40 mV and 33.6±1.7 ms at +40 mV in the absence of drug and 216.0±17.2 ms at –40 mV and 40.9±1.0 ms at +40 mV in the presence of (S)-2, *n* = 7, P = 0.89) but significantly increased the slow component at all potentials tested (P<0.05–P<0.001, *n* = 7). The τ_slow_ at +40 mV was 288.0±33.3 ms and 664.8±71.8 ms in the absence and presence of (S)-2, respectively. The deactivation kinetics of Kv7.2-W236L were not affected by (S)-2 (data not shown).

### (S)-2 Sensitivity of Heteromeric Kv7.4/Kv7.4-W242L Channels

**Figure 9:**
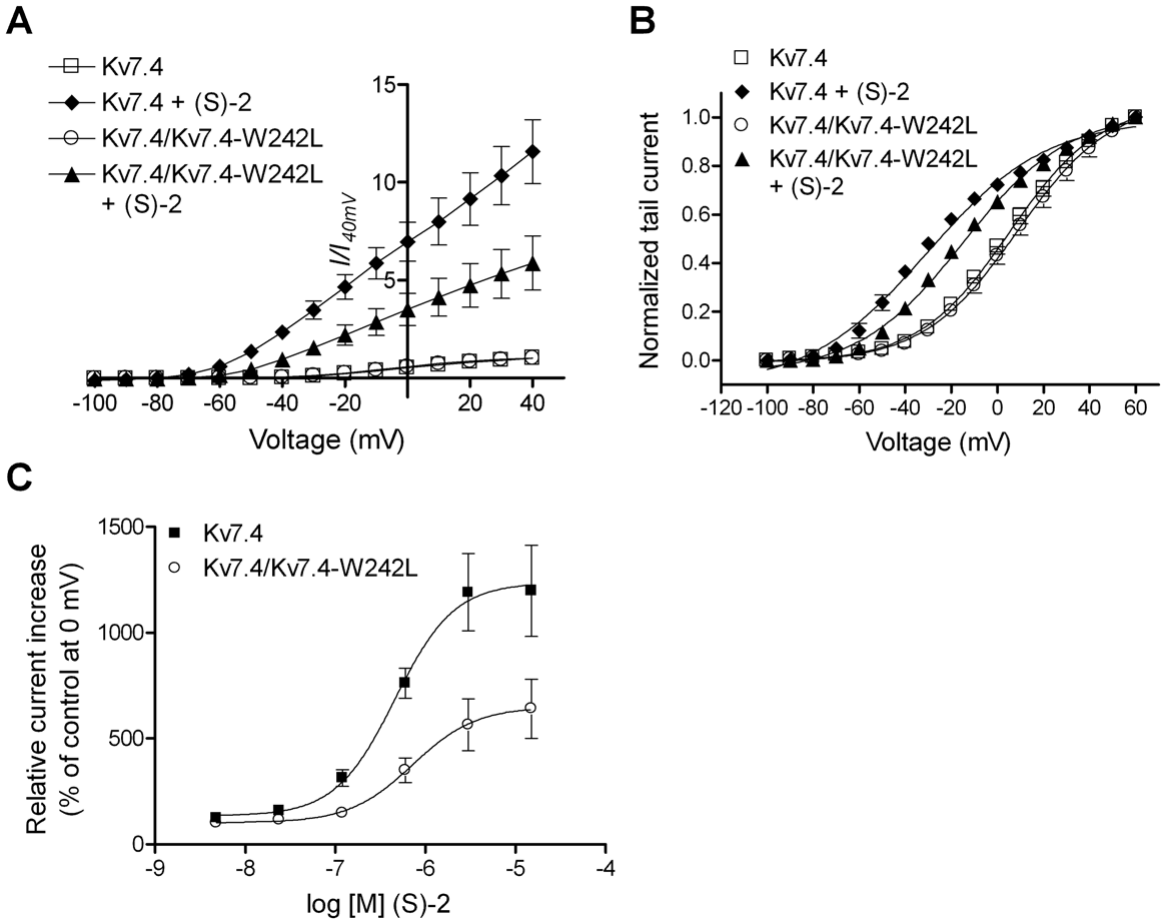
Sensitivity of heteromeric Kv7.4/Kv7.4-W242L channels to (S)-2. (A) Current-voltage (I-V) relationship of Kv7.4 and Kv7.4/Kv7.4-W242L in the absence and presence of 15 µM (S)-2. The steady state peak current measured at potentials between –100 and +40 mV were normalized against the current at +40 mV in control recordings and plotted against the test potential. (B) Effect of (S)-2 on the voltage-dependence of activation of Kv7.4 and Kv7.4/Kv7.4-W242L. Tail currents measured after stepping back to –120 mV from potentials between –100 and +60 mV in the absence and presence of 15 µM (S)-2 were normalized and plotted against the preceding potential. The tail current-voltage relationship was then fitted to the Boltzmann equation to yield half-activation potentials (V_0.5_): Kv7.4 (5.1±1.3 mV); Kv7.4+(S)-2 (−30.5±1.6 mV); Kv7.4/Kv7.4-W242L (4.1±1.6 mV); Kv7.4/Kv7.4-W242L+(S)-2 (−15.0±1.0 mV). (C) Dose-response relationship of (S)-2 on Kv7.4 and Kv7.4-W242L. The steady state peak currents elicited by a 5 s step to 0 mV in response to increasing concentrations of (S)-2 were normalized to the current in the absence of compound and plotted as a function of the concentration of (S)-2. The values were then analyzed by non-linear regression to fit a sigmoidal curve. The EC_50_ values were determined to 0.46 µM and 0.72 µM and the Hill coefficients to 1.32±0.17 and 1.24±0.25 for Kv7.4 and Kv7.4/Kv7.4-W242L, respectively. Bars represent S.E.M. and *n* = 6–10.

Retigabine is proposed to be dependent on the critical tryptophan residue being present in all four subunits of a channel complex to exert its effects [24]. To address whether this is also the case for (S)-2 we co-expressed WT Kv7.4 and Kv7.4-W242L in a ratio of 1∶1 in oocytes. If the activity of (S)-2 was dependent on it being bound to all four subunits, we would expect the increase in current after application of (S)-2 to correspond to the population of homomeric Kv7.4 channels present. Assuming random assembly, the binomial distribution predicts this population to be 6.25% of the total. As seen in figure 9A, the increase in current after exposure of oocytes co-expressing WT and mutated Kv7.4 channels to 15 µM (S)-2 was much larger than to be explained solely as an effect of the compound on the small percentage of pure WT channels that would be present. Both the increase in current amplitude (1158.1±147.9% of control for Kv7.4 and 587.1±147.9% of control for Kv7.4/Kv7.4-W242L at +40 mV) and the shift in V_0.5_ (−35.6 mV for Kv7.4 and –19.1 mV for Kv7.4/Kv7.4-W242L) was approximately half of that observed when stimulating homomeric Kv7.4 with 15 µM (S)-2 (fig. 9A and B). An explanation for these observations could be that the WT and W242L mutant subunits were not forming heteromers. To test this we co-expressed Kv7.4-W242L with the dominant negative pore mutant Kv7.4-G285S [8]. Oocytes co-expressing Kv7.4-W242L and Kv7.4-G285S did not give rise to any measurable current (data not shown), suggesting assembly of the two mutants. We concluded that the W242L mutant was capable of forming heteromers with WT subunits.

To test whether the effect of implementing mutated subunits in the channel complex was to reduce the potency of the compound we compared dose-response curves of (S)-2 on homomeric Kv7.4 and heteromeric Kv7.4/Kv7.4-W242L channels (fig. 9C). Such a reduction in potency could imply that the compound bound in a cooperative fashion, i.e. binding to one subunit would facilitate the binding to another. These experiments showed that the presence of Kv7.4-W242L subunits does not change the compound's potency. The EC_50_ values for homomeric Kv7.4 and heteromeric Kv7.4/Kv7.4-W242L channels were 0.35 µM (pEC_50_ = 6.45±0.09 M) and 0.66 µM (pEC_50_ = 6.18±0.04 M), respectively (*n* = 8). Although there was a statistically significant difference between the pEC_50_ values (P = 0.01) the difference is likely too small to be of importance. There was no significant difference between the Hill coefficient for (S)-2 on homomeric Kv7.4 and heteromeric Kv7.4/Kv7.4-W242L channels (1.68±0.3 for Kv7.4 and 1.41±0.17 for Kv7.4/Kv7.4-W242L, P>0.4) indicating no change in cooperativity.

## Discussion

Because of the important role Kv7 channels play in controlling electrical homeostasis in the brain, increasing Kv7 channel activity will generally reduce neuronal excitability. Since the identification of the Kv7 channels in the late 1990's, several compounds have been described as openers or inhibitors of the channels. However, only a few of these compounds have yet earned a thorough examination.

The acrylamide (S)-2 has previously been described as a potent opener of murine Kv7.2 channels and has demonstrated significant activity in reducing neuronal hyperexcitability in rat hippocampal slices [19]. Here we show that (S)-2 activates human homomeric Kv7.2-5 and heteromeric Kv7.2/3 channels expressed in *Xenopus laevis* oocytes. In contrast to the effect on the neuronal Kv7 channels (S)-2 weakly inhibits homomeric Kv7.1 channels. As Kv7.1 together with KCNE1 constitutes the molecular correlate of the slow repolarising current in the heart (the I_Ks_ current), this observation could indicate safety liability and potential for inducing torsades de pointes [25]. Yet, several Kv7.1 channel active compounds exhibit markedly reduced activity when Kv7.1 is co-expressed with KCNE1 [20], [26], [27]. This, however, remains to be tested for (S)-2.

Similar to most Kv7 channel openers described, (S)-2 affected the kinetics of the channels, although the effect was different from that of other compounds [17], [20], [28], [29]. Interestingly, as with the effect of the compound on the current amplitude of Kv7.2 there was a crossover in the effect of (S)-2 on τ_slow_ resulting in a significant increase in this time constant at more positive potentials. This could indicate that the two effects are related and that the reduction in current amplitude at high potentials results from an increase in τ_slow_. For Kv7.4, which exhibited monoexponential activation kinetics, τ was significantly reduced in the presence of (S)-2 at all potentials tested.

Application of (S)-2 resulted in large effects on deactivation kinetics of Kv7.2. Intriguingly, for Kv7.4, the effect of (S)-2 on deactivation kinetics was more dramatic increasing both τ_fast_ and τ_slow_ and strikingly changing the contribution of the slow component of the deactivation kinetics. Notably, at some potentials the deactivation was not only slowed but the kinetic mode was completely altered. Slowing of deactivation may have profound effects on cellular excitability, as the channels with (S)-2 bound will remain open for a longer period each time they are activated. Hence, (S)-2 appears especially well suited for further studies of the impact of Kv7 channel deactivation rates on various physiological parameters.

Inactivation has recently been described as a new regulatory mechanism for Kv7.4 and Kv7.5 [21], [22]. At physiologically relevant membrane potentials, the current is reduced by more than 30% due to steady-state inactivation. We found that (S)-2 reduced the inactivation of Kv7.4. One consequence of reduced inactivation would be increased current amplitudes; however, the effect of (S)-2 on the current amplitude of Kv7.4 was much larger than what can be accounted for by reduced inactivation alone.

There is evidence indicating that Kv7 channels only display one conductance level [30]–[33]. Neither of the drugs which effect has been examined at the single-channel level alters the conductance of the channels but instead increases the open probability [31]–[33]. It is likely that (S)-2 also works by increasing the open probability of the neuronal Kv7 channels.

A conserved tryptophan residue within the fifth transmembrane segment of Kv7.2-5 is a structural element of a major binding site in the neuronal Kv7 channels. Retigabine, BMS-204352 and (S)-1 are all dependent on the residue for their effects [20], [23], [24]. Interestingly, all of these tryptophan-dependent compounds behave similarly regarding effects on current amplitude and voltage-dependence of activation, albeit with different potency and efficacy. Recently, several groups indicated that Zink pyrithione, meclofenamic acid and diclofenac recognize other binding sites [28], [33], [34].

We found that (S)-2 also is dependent on the tryptophan residue for its enhancing effect, indicating an overlapping pharmacophore with retigabine. However, it is likely that besides the tryptophan residue, (S)-2 interacts with other residues than retigabine, as (S)-2 modulates the inactivation of Kv7.4, an ability retigabine does not possess [21]. The inactivation of the Kv7.4-W242L mutant was not affected by (S)-2, indicating that the integrity of the whole pharmacophore is crucial also for (S)-2 modulation of inactivation.

Intriguingly, substitution of the tryptophan residue in Kv7.2 with leucine (Kv7.2-W236L) produced a channel that was inhibited by (S)-2 seen as a reduction in current amplitude without affecting the voltage-dependence of activation. In addition, the slow time constant of activation was increased. We propose that the mechanism behind this inhibition is the same as for the secondary inhibitory action of (S)-2 observed on the current amplitude and τ_slow_ of WT Kv7.2. In fact, plotting the current amplitude of Kv7.2 and Kv7.2-W236L in the presence of (S)-2 against the voltage above their resulting threshold yields two superimposed curves. Hence, considering the (S)-2-induced shift in voltage-dependence of activation, it can be argued that (S)-2 decreases the current amplitude of Kv7.2 at all potentials relative to the threshold of activation. The effect of (S)-2 on Kv7.2 may thereby be divided into two entities; an activating part visible as shift in voltage-dependence of activation and an inhibitory part visible as a decrease in current amplitude and an increase in τ_slow_. Apparently, the W236L mutation only disables the (S)-2-induced activation, thereby unmasking the inhibitory component of the drug effect. Since inhibition requires higher concentrations than activation (fig. 4A vs. fig. 8F) it is likely that these effects rely upon action at different interactions sites, as has been suggested for the secondary inhibitory effect of retigabine [18], [24], [31]. It is also possible that the activating and inhibitory effects are consequences of the compound binding in two different conformations to an overlapping site. Interestingly, Kv7.4-W242L was not inhibited by (S)-2, indicating a degree of subtype selectivity.

It has been demonstrated that four tryptophan residues within a tetramer are necessary for activation of Kv7 channels by retigabine [24]. We present data indicating that this may not be the case for the activation of Kv7 channels by (S)-2. Our results do not fit to a model where the channel responds to (S)-2 in an “all-or-non” fashion. One plausible explanation is that the effect of (S)-2 is proportional to the number of subunits that are occupied by the compound. To assess our hypothesis the effect of (S)-2 on each stoichiometry of heteromeric Kv7.4/Kv7.4-W242L channels needs to be determined at the single-channel level. Albeit, this approach is hampered by the low open probability of Kv7.4 channels [32].

In conclusion, (S)-2 is a modulator of Kv7 channels with differential effects on the channel subtypes. We present data revealing that the consequence of binding is not purely activating for all neuronal subtypes, suggesting the existence of a subtype specific additional inhibitory binding site or a mode of binding not dependent on the described tryptophan residue. Improved understanding of those molecular determinants may contribute to the design of subtype selective channel modulators. Furthermore, our pharmacological profiling suggests that (S)-2 is a suitable tool complementing retigabine in the study of Kv7 channels.
